# The impact of post-stroke pneumonia on survival and functional outcomes: A systematic review and meta-analysis

**DOI:** 10.12669/pjms.41.10.12916

**Published:** 2025-10

**Authors:** Youguang Weng, Ting Shen

**Affiliations:** 1 Youguang Weng Department of Critical Care Medicine, Huzhou Nanxun People’s Hospital, Huzhou, Zhejiang, 313009, China; 2 Ting Shen Department of Pharmacy, Huzhou Nanxun People’s Hospital, Huzhou, Zhejiang, 313009, China

**Keywords:** Stroke-associated pneumonia, Mortality, Functional outcomes, Meta-analysis

## Abstract

**Objective:**

To evaluate the impact of stroke-associated pneumonia (SAP) on mortality risk and poor functional outcomes.

**Methodology:**

PubMed, Embase and Scopus databases were searched to identify studies with adult stroke patients (≥40 years) who were diagnosed with SAP. Risk of mortality and poor functional outcome were of primary interest. Results were reported as pooled odds ratios (OR) or hazard ratios (HR) with 95% confidence intervals (CI).

**Results:**

A total of 22 studies were included. SAP was associated with the increased risk of in-hospital mortality (OR 2.67, 95% CI: 1.82, 3.93) and mortality at one (OR 2.11, 95% CI: 1.17, 3.79), two (OR 6.97, 95% CI: 4.63, 10.5) and three months (OR 4.26, 95% CI: 2.44, 7.44) of follow up compared to stroke patients without SAP. Risk of mortality at ≥1 year of follow up was also higher in patients with SAP (HR 2.44, 95% CI: 1.33, 4.48). SAP also correlated with the increased risk of poor functional outcome (OR 4.82, 95% CI: 3.47, 6.69)

**Conclusion:**

SAP is associated with significant adverse impact on survival and functional outcomes of stroke patients and further strengthen the need for a comprehensive approach to stroke management.

***Registration No:** PROSPERO [CRD420251020683]*.

## INTRODUCTION

Stroke is one of the leading cause of mortality globally and an important cause of premature death and disability.^1^,^2^ Medical and neurological complications, such as pneumonia, are considered primary contributors to post-stroke mortality.^3^,^4^ Stroke-related pneumonia (SAP) that affects 4% to 32% of stroke patients has a profound impact on both early and late mortality,^5^ and is associated with a significant financial burden.^6^,^7^

Studies attribute SAP to post-stroke immunodepression, aspiration of oral secretions and oral intake in the presence of dysphagia.^8^,^9^ SAS risk factors include the degree of neurological impairment, advanced age and pre-existing comorbidities,^10^,^11^ and the severity of stroke has been consistently linked to an elevated risk of SAP.^12^,^13^

Acute stroke leads to a cascade of changes in peripheral immune responses, that manifest as transient lymphopenia and monocyte deactivation^14^ which make stroke patients more susceptible to infections. However, the intricate interplay between stroke and the immune system extends beyond these systemic effects, influencing respiratory mechanisms that significantly contribute to the development of pneumonia. Stroke-induced immunomodulation impacts tracheal epithelium, leading to a reduction in pulmonary clearance and impaired secretion drainage^15^,^16^ that, subsequently, promote initiation and progression of pneumonia. There is currently no systematic review and meta-analysis that summarized available studies to investigate the impact of SAP on the risk of mortality and functional disability in patients with stroke. This meta-analysis aimed to fill this gap by examining existing literature and pooling their individual findings to determine if SAP is associated with adverse survival and functional outcomes in this group of patients.

## METHOOLOGY

### Inclusion and exclusion criteria:

Eligibility criteria were based on PECOS format. We incorporated studies involving adult (≥40 years) patients who were diagnosed with stroke, irrespective of the type or the underlying aetiology (*Population*). *Exposure* and *Comparison* was patients with post-stroke pneumonia and without post-stroke pneumonia. The *Outcome* of interest was the risk of mortality and poor functional outcomes. Outcomes were not predefined and all definitions were acceptable.

### Study types:

Cohort studies, case-control studies and studies based on secondary analyses of clinical trial data were included. Eligible studies had to be English-language and peer-reviewed. Studies involving paediatric patients, studies lacking a clear diagnosis of post-stroke pneumonia and studies with insufficient data or unclear reporting of outcome were excluded. Additionally, review articles, editorials, letters, commentaries and case reports, were also excluded.

### Search strategy:

Comprehensive literature search was done by two reviewers (YS, TW) across PubMed, Embase and Scopus databases to identify studies aligning with the research objectives. The search strategy encompassed a combination of key terms: (“post-stroke pneumonia”) AND (“survival outcomes” OR “mortality” OR “death rates” OR “life expectancy”) AND (“functional outcomes” OR “disability” OR “functional impairment” OR “quality of life”). The search query was modified for each database. Studies published between January 1, 2000 until January 31, 2024, were considered. In addition to electronic searches, manual screening of reference lists and review articles was carried.

### Identifying studies for inclusion:

Studies, identified by the initial electronic search, were deduplicated. Titles and abstracts of the remaining studies were assessed by the two study authors for relevance. Studies that met the initial criteria underwent a detailed full-text evaluation. Discrepancies or disagreements were resolved by discussions.

### Quality assessment:

Quality of the selected studies was assessed using the Newcastle-Ottawa Scale (NOS).^17^ This structured framework involves a systematic evaluation of studies, considering key criteria such as the selection of study groups, comparability between groups and the ascertainment of outcomes. The maximum achievable score is nine, scores >8 meant high, 6-7 meant medium, and <6 meant low study quality.

### Data extraction:

The extraction of data was conducted by two independent authors using a standardized form that included variables reflective of study identifiers such as authors, publication year, study location as well as study design, subject characteristics, type of stroke, definition of poor functional outcome adopted, sample size and key findings. To ensure accuracy and consistency, any disagreements between the two authors during the data extraction process were resolved through thorough discussions and consensus.

### Statistical analysis:

Analysis was done using STATA version 15.0. Pooled effect sizes were presented as odds ratios (OR) or hazard ratios (HR) along with 95% confidence intervals (CI). Subgroup analyses were conducted based on study design, location, type of stroke and sample size. The random-effects model was employed. I^2^ > 50% represented significant heterogeneity among the included studies.^18^ To assess publication bias, Egger’s test and funnel plots were utilized.^19^ The protocol was officially registered in PROSPERO [CRD420251020683]. Preferred Reporting Items for Systematic Reviews and Meta-Analyses (PRISMA) guidelines were adhered.^20^

## RESULTS

**Fig.1:**
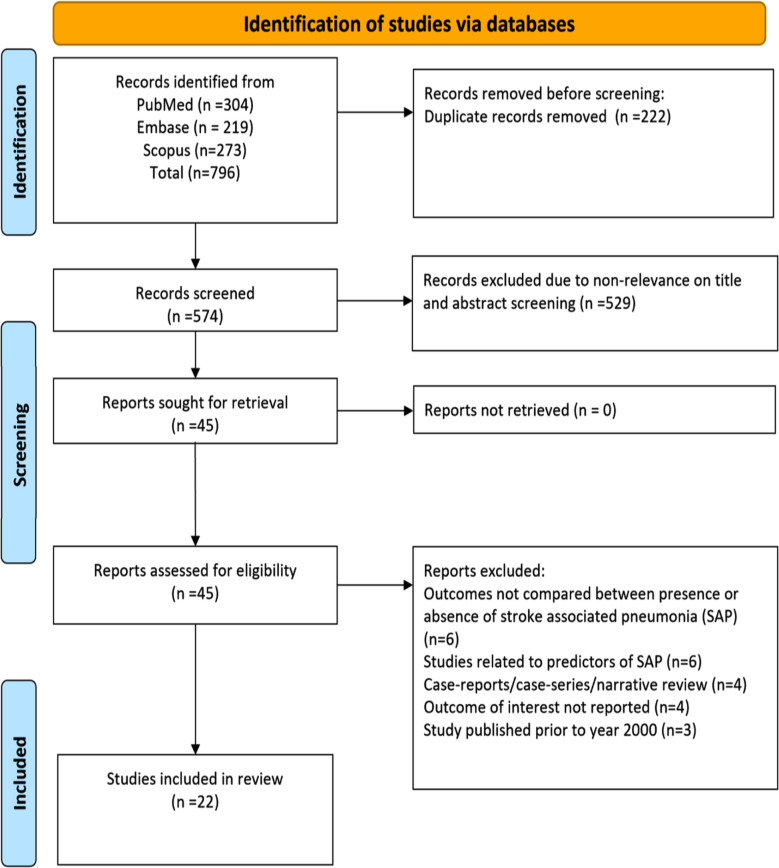
PRISMA flowchart.

A total of 796 studies were detected by the search across three databases. Ultimately, 22 studies with 51,64,020 patients were included in the final analysis. Fig.1^11^,^21^-^41^ Of them, 14 studies were retrospective cohort studies and seven studies had a prospective cohort design. One study used secondary analysis of data collected as part of the clinical trial. Most of the studies included patients with ischaemic stroke. Included studies were similar in terms of outcome reporting, such as functional outcome that was assessed using the modified Rankin Scale score (mRS). However, the operational definition of the exposure of interest i.e., SAP, differed among the studies. While some studies defined SAP as new antibiotic initiation for suspected pneumonia within the first seven days of admission of stroke, others based it on International Classification of Diseases, clinical modification codes (ICD-9-CM). Studies also defined SAP as a medical complication that happened within the first 30 days after the stroke and was confirmed by radiography. In some studies, the diagnosis was based on clinical, radiological and biochemical parameters such as persistent infiltrations on chest x-ray accompanied by leukocytosis with left shift or leukocytopenia, at least 50% increase in the levels of C-reactive protein, hypo- (<36.5 ^0^C) or hyperthermia (>38.5 ^o^C), cough with purulent sputum or rattling noises on auscultation (Supplementary Table-I). On quality assessment based on NOS, there were 10 studies with a score of 8, 10 studies with a score of 7 and two studies with a score of 6.

**Supplementary Table-I:** Details of the included studies.

Author	Study design; Region	Participant characteristics and type of stroke	Functional outcome definition	Definition of stroke-associated pneumonia (SAP)	Sample size	NOS score
Lobo Chaves et al (2023)^21^	Retrospective cohort; United Kingdom	Mean age of around 80 years; female (52%) Type of stroke, not provided	Poor functional outcome: modified Rankin Scale (mRS≥3)	New antibiotic initiation for suspected pneumonia within the first 7 days of admission	1,99,561	S-4 C-2 O-2
Zhang et al (2023)^22^	Retrospective cohort; China	Mean age around 66 years; male (65%) Ischemic stroke	Poor functional outcome: modified Rankin Scale (mRS≥3)	Pneumonia diagnosed within the first 7 days of admission after stroke onset	248	S-4 C-2 O-1
Gittins et al (2023)^23^	Retrospective cohort; United Kingdom	Majority aged 60 years and above (85%); female (50%) Ischemic stroke (88%)	Not applicable	New antibiotic initiation for pneumonia within the first 7 days of stroke admission	3,39,139	S-4 C-2 O-2
Schumann-Werner et al (2023)^24^	Retrospective cohort; Germany	Mean age 74 years; females (48%) Ischemic stroke	Poor functional outcome: modified Rankin Scale (mRS≥3)	Based on the modified Center for Disease Control and Prevention (CDC) criteria; diagnosis required to be made within the first 7 days of admission after stroke onset	420	S-4 C-2 O-1
Gonçalves-Pereira et al (2023)^25^	Retrospective cohort; Portugal	Adults older than 60 years; males (51%); Ischemic stroke (85%)	Not applicable	Based on International Classification of Diseases, clinical modification codes (ICD-9-CM)	96038	S-4 C-1 O-1
de Jonge et al (2022)^26^	Retrospective cohort; Netherlands	Median age 71 years; males (56%) Ischemic stroke (89%)	Poor functional outcome: modified Rankin Scale (mRS≥3)	Lower respiratory tract infections diagnosed within 90 days of admission after stroke onset	10821	S-4 C-2 O-2
Huang et al (2022)^27^	Prospective cohort; China	Median age of around 71 years; male (54%) Ischemic stroke	Poor functional outcome: modified Rankin Scale (mRS≥3)	Diagnosis based on the 2015 diagnostic criteria from the PISCES group	776	S-4 C-2 O-1
Zhao et al (2021)^28^	Prospective cohort; China	Mean age of 55 years; male (48%) Haemorrhagic stroke (90%)	Not applicable	Based on the modified Center for Disease Control and Prevention (CDC) criteria	200	S-4 C-2 O-1
Yuan et al (2021)^11^	Prospective cohort; China	Majority aged older than 65 years (57%); males (65%) Ischemic stroke	Poor functional outcome: modified Rankin Scale (mRS score of 2-6)	According to the recommendations published by the Pneumonia in Stroke Consensus Group	451	S-4 C-2 O-2
Prust et al (2021)^29^	Prospective cohort; Zambia	Mean age 60 years; females (61%) Ischemic stroke (55%)	Not applicable	Participants were categorized as having SAP if ≥ 4 of these Variables: fever (temperature >38°C), tachypnea (respiratory rate >20), hypoxemia (oxygen saturation <92%), cough, rhonchi, witnessed aspiration event and initiation of antibiotics for clinically suspected pneumonia	125	S-4 C-2 O-1
Patel et al (2020)^30^	Retrospective cohort; USA	Mean age 72 years; females (53%) Ischemic stroke	APRDRG (All Patients Refined Diagnosis Related Groups) risk of mortality and severity assigned using software developed by 3M Health Information Systems [ score 1 indicates minor, 2—moderate, 3—major, 4—the extreme likelihood of risk of death and loss of function, respectively]	Based on International Classification of Diseases, clinical modification codes (ICD-9-CM)	42,24,924	S-4 C-2 O-2
Zhu et al (2020)^31^	Retrospective cohort; China	Mean age around 62 years; male (68%) Ischemic stroke	Poor functional outcome: modified Rankin Scale (mRS≥3)	Lower respiratory tract infections diagnosed within the first 7 days of admission after stroke onset	112	S-4 C-2 O-1
Montmollin et al (2019)^32^	Prospective cohort; France	Median age of 69 years; male (68%) Ischemic stroke	Poor functional outcome: modified Rankin Scale (mRS≥4)	Pneumonia diagnosed within the first 7 days of admission after stroke onset	195	S-4 C-2 O-1
Schwarz et al (2018)^33^	Retrospective cohort; Australia	Mean age of 70 years; males (45%); Ischemic stroke	Not applicable	Based on International Classification of Diseases, clinical modification codes (ICD-9-CM)	110	S-4 C-1 O-1
Teh et al (2018)^34^	Retrospective cohort; United Kingdom	Mean age of around 77 years; female (53%) Ischemic stroke (87%)	Poor functional outcome: modified Rankin Scale (mRS≥3)	Pneumonia diagnosed on admission or within the first 7 days of admission after stroke onset	9238	S-4 C-2 O-2
Colbert et al (2016)^35^	Retrospective cohort; USA	Subjects aged >60 years (80%); male (48%) Ischemic stroke	Not applicable	Based on International Classification of Diseases, clinical modification codes (ICD-9-CM)	74722	S-4 C-2 O-1
Kalra et al (2016)^36^	Secondary analysis of RCT data; United Kingdom	Mean age 78 years; females (57%) Ischemic stroke (90%)	Poor functional outcome: modified Rankin Scale (mRS≥3)	Based on the Center for Disease Control and Prevention (CDC) criteria	1217	S-4 C-2 O-2
Yu et al (2016)^37^	Prospective cohort; Taiwan	Mean age 70 years; females (45%) Ischemic stroke	Not applicable	Based on new or persistent infiltrations on chest x-ray in combination with leukocytosis with left shift or leukocytopenia, at least 50% increase of C-reactive protein compared to baseline, hypo- (<36.5^0^C) or hyperthermia (>38.5 ^o^ C), cough with purulent sputum or characteristically rattling noises on auscultation	956	S-4 C-2 O-2
Wilson et al (2012)^38^	Retrospective cohort; USA	Mean age 70 years; females (55%) Both ischemic and haemorrhagic stroke (proportion not mentioned)	Not applicable	Based on International Classification of Diseases, clinical modification codes (ICD-9-CM)	1,83,976	S-4 C-2 O-1
Finlayson et al (2011)^39^	Retrospective cohort; Canada	Mean age 72 years; males (52%) Ischemic stroke	Poor functional outcome: modified Rankin Scale (mRS≥3)	Defined as a radiographically confirmed medical complication that occurred within the first 30 days after the stroke onset.	8251	S-4 C-2 O-1
Hong et al (2008)^40^	Prospective cohort; Republic of Korea	Mean age 66 years; males (56%) Ischemic stroke	Poor functional outcome: modified Rankin Scale (mRS≥3)	Based on crackle and fever with radiographic evidence and/or purulent sputum requiring antibiotics	1254	S-4 C-2 O-1
Katzan et al (2003)^41^	Retrospective cohort; USA	Mean age 77 years; females (42%) Both haemorrhagic and ischemic stroke	Not applicable	Based on International Classification of Diseases, clinical modification codes (ICD-9-CM)	11286	S-4 C-2 O-2

RCT, randomised controlled trial; NOS, Newcastle Ottawa Scale; S, selection; C, comparability; O, outcome assessment; ICD, international classification of diseases.

**Table-I:** Subgroup analysis of outcomes.

Subgroups	Risk of in-hospital mortality	Risk of mortality at 3 months	Risk of mortality at 1 year of more	Risk of poor functional outcome
	Pooled odds ratio (OR) with 95% CI (Number of studies; I^2^)	Pooled odds ratio (OR) with 95% CI (Number of studies; I^2^)	Pooled Hazard ratio (HR) with 95% CI (Number of studies; I^2^)	Pooled odds ratio (OR) with 95% CI (Number of studies; I^2^)
***Study design***				
Prospective cohort	1.47 (0.37, 5.83) (2; 86.0%)	9.68 (4.53, 20.7) (2; 45.3%) *	3.07 (0.74, 12.8) (2; 95.5%)	5.27 (2.78, 9.98) (5; 76.4%) *
Retrospective cohort	2.96 (1.93, 4.54) (12; 99.8%) *	2.77 (1.90, 4.06) (4; 45.9%) *	1.99 (1.01, 4.48) (2; 96.3%) *	4.72 (3.09, 7.19) (8; 99.6%) *
***Type of stroke***				
Ischaemic	2.89 (1.77, 4.72) (13; 99.6%) *	4.26 (2.44, 7.44) (7; 89.9%%) *	2.44 (1.33, 4.48) (4; 94.2%) *	5.14 (4.08, 6.49) (13; 77.0%) *
***Sample size***				
≥500	2.57 (1.65, 4.00) (10; 99.9%) *	4.36 (2.11, 9.01) (4; 94.7%) *	2.86 (1.34, 6.13) (3; 95.7%) *	5.57 (3.79, 8.19) (9; 99.5%) *
<500	3.38 (1.05, 10.9) (5; 86.1%) *	4.07 (2.18, 7.6) (3; 0.0%) *	1.49 (1.01, 2.20) (1; ---) *	3.54 (1.55, 8.12) (5; 78.2%) *
***Location***				
Asia	2.66 (1.56, 4.55) (94%)*	-	-	4.16 (2.22, 7.64) (89.9%)*
Europe	2.44 (1.33, 4.48) (88%)*			4.46 (1.21, 16.39) (77%)*
North America	3.29 (1.37, 7.91) (99%)*			4.46 (2.65, 7.56) (99%)
Others	12.21 (4.08, 36.51) (99%)			

*indicates statistical significance at p<0.05.

### Risk of mortality:

**Fig.2:**
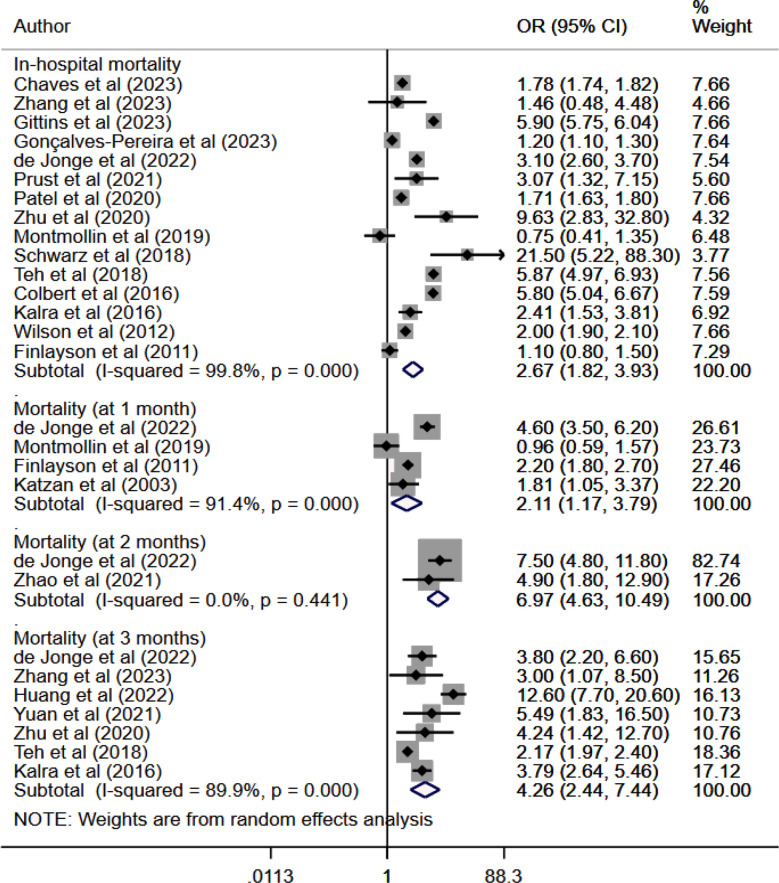
Meta-analysis of short-term mortality among those with and without stroke associated pneumonia.

**Fig.3:**
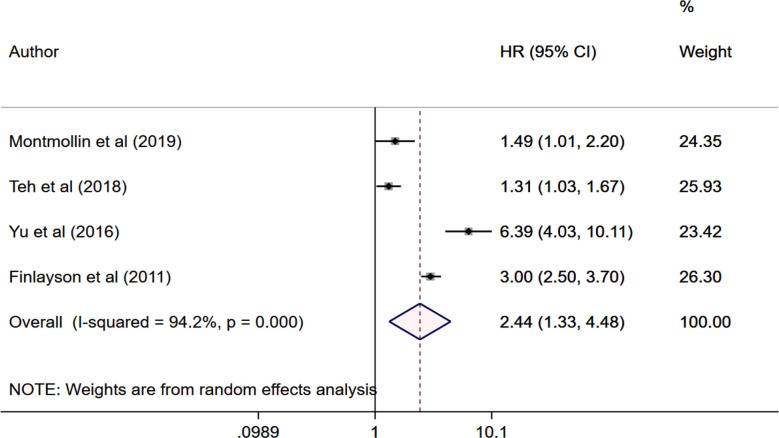
Meta-analysis of mortality at one year or more of follow up among those with and without stroke associated pneumonia.

In stroke patients, SAP correlated with significantly higher risk of in-hospital mortality (OR 2.67, 95% CI: 1.82, 3.93; n=15, I2=99.8%), mortality at one month (OR 2.11, 95% CI: 1.17, 3.79; n=4, I2=91.4%), two months (OR 6.97, 95% CI: 4.63, 10.5; n=2, I2=0.0%) and three months (OR 4.26, 95% CI: 2.44, 7.44; n=7, I2=89.9%) of follow up compared to patients without SAP.Fig.2 The risk of mortality at ≥1 year of follow up was also higher in patients with SAP (HR 2.44, 95% CI: 1.33, 4.48; n=4, I2=94.2%). Fig.3 There was no evidence of publication bias on Egger’s test (p>0.05) or funnel plot (Supplementary Fig.1).

**Figure:**
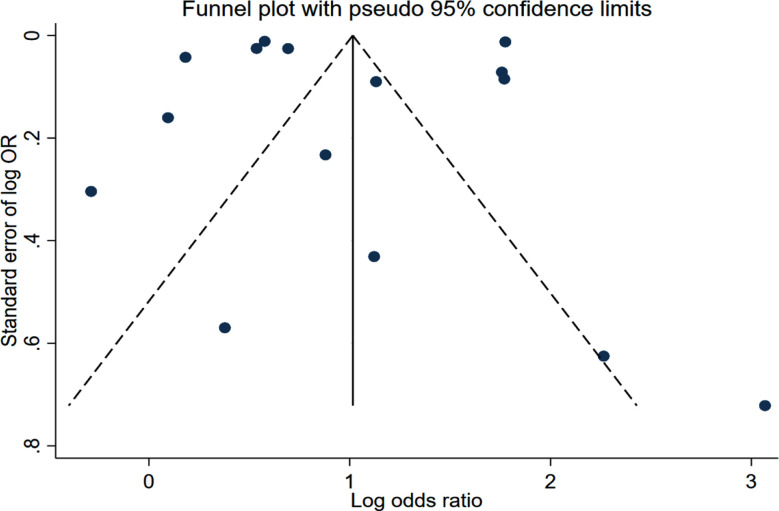
Supplementary Fig.1.

Subgroup analysis revealed that studies with a retrospective design consistently showed elevated risk of mortality across all considered time points, whereas studies with a prospective cohort design reported comparable risk of mortality in both groups (Table-I). Subgroup analysis of the cohort of patients with ischaemic stroke indicated elevated risk of mortality across all time points. These findings remained consistent regardless of whether the studies had small (<500) or large sample sizes (≥500) and irrespective of location (Table-I).

### Risk of poor functional outcomes:

**Fig.4:**
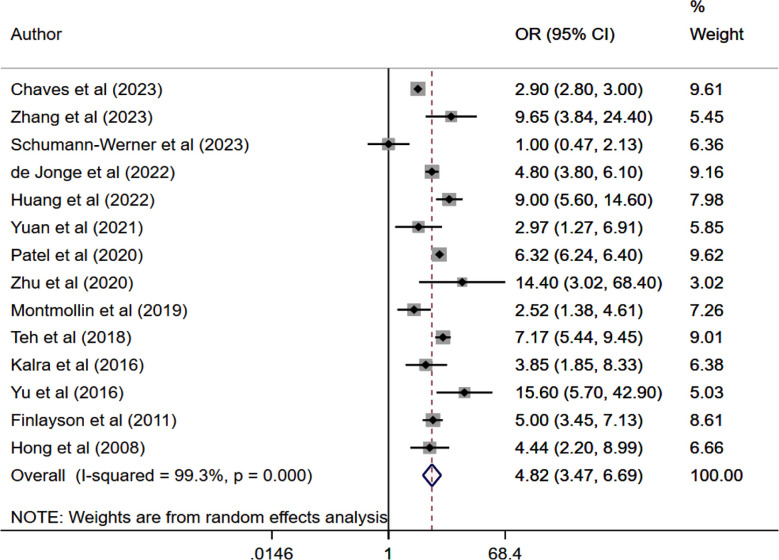
Meta-analysis of poor functional outcome among those with and without stroke associated pneumonia.

Patients with SAP had increased risk of poor functional outcome (OR 4.82, 95% CI: 3.47, 6.69; n=14, I2=99.3%). (Fig.4) (with no evidence of publication bias on Egger’s test (p>0.05) or funnel plot (Supplementary Fig.2). Subgroup analysis showed that the association between SAP and an elevated risk of poor functional outcomes remained unchanged across various subgroups, i.e., those categorized by study design, type of stroke, location and sample size (Table-I).

**Figure:**
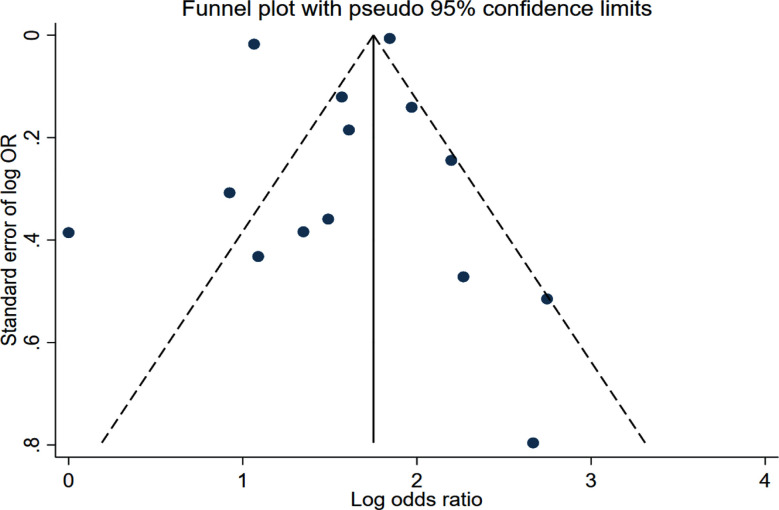
Supplementary Fig.2.

## DISCUSSION

This study further emphasizes the critical impact of SAP on both survival and functional outcomes. Our results demonstrated increased risk of mortality in patients who developed pneumonia in the aftermath of a stroke. This increased risk of mortality may be due to a complex array of pathophysiological mechanisms, such as post-stroke immunodepression, a state marked by a transient suppression of immune function^42^ that renders patients more susceptible to pneumonia. Neurological impairments resulting from a stroke, particularly dysphagia, may also contribute significantly to poorer outcomes in this population of patients.^42^,^43^ Dysphagia increases the risk of aspirating oral secretions into the lungs and subsequent development of pneumoni.^43^ Additionally, stroke-induced changes in the tracheal epithelium, coupled with impaired mucociliary clearance and diminished cough reflex, impact effective clearance of pathogens from the respiratory tract.^44^,^45^

On a more systemic level, the inflammatory response triggered by post-stroke pneumonia may potentially lead to multi-organ dysfunction.^46^ The cardiovascular strain imposed by pneumonia exacerbates the already compromised cardiovascular and respiratory systems, further increasing the risk of mortality.^47^,^48^ Additionally, secondary complications, such as the exacerbation of preexisting conditions and delayed rehabilitation due to pneumonia, might also contribute to the overall higher mortality risk in stroke patients with SAP.

Our analysis revealed a significant association between pneumonia and poor functional outcomes, indicating a substantial impact on the ability of stroke survivors to regain independence and adequate quality of life. We may speculate that one key factor, contributing to this increased risk, may be the systemic inflammatory response triggered by pneumonia.^49^ The influx of inflammatory mediators exacerbates the existing neuroinflammation from the stroke, potentially amplifying neuronal damage and negatively impacting the intricate process of neuroplasticity that is essential for recovery.^14^,^50^ Furthermore, compromised respiratory muscle strength due to pneumonia can affect the ability to perform daily living activities, further adding to functional limitations of stroke survivors.^51^ Pneumonia-induced physical deconditioning and fatigue might slow the rehabilitation process, hindering the progress of recovery and limiting the overall success of rehabilitation interventions.

The inter-study heterogeneity was high in all our meta-analysis and hence, the results must be interpreted with caution. We attempted to explore the source of heterogeneity by multiple subgroup analyses, however, even in those subgroups, the heterogeneity remained high indicating other factors at play. A detailed subgroup analyses was not possible for several confounders like SAP definition and stroke severity either due to heterogeneous groups or limited data. The definition of SAP can skew results as SAP defined by one definition may be missed by another. Without a homogenous definition, the generalizability of the summary estimated is also significantly reduced. Another major difference in definitions was that of poor functional outcomes. Some studies defined it as mRS ≥3 while others used the cut-off of mRS ≥4. Such inconsistency may also result in biased pooled estimates. Moreover, it is plausible that several unmeasured confounders may have influenced the study results. Given the high inter-study heterogeneity, the pooled estimates may not be entirely reliable and hence should be supported by future studies.

### Clinical implications:

The recognition of SAP as a significant contributor to increased mortality and poor functional outcomes has significant clinical implications for the management and care of stroke patients. First and foremost, healthcare providers need to be vigilant in identifying and promptly addressing respiratory complications. Preventive measures such as early mobilization, dysphagia screening and pulmonary care protocols are needed. Optimizing rehabilitation strategies, including respiratory exercises, may play a pivotal role in mitigating the impact of pneumonia on functional outcomes. Moreover, our findings underscore the possible importance of immunomodulatory interventions to improve the immune response during the post-stroke period, potentially reducing patients’ susceptibility to infections.

### Future research directions:

Future research should delve focus on the specific aspects of SAP to refine preventive and therapeutic strategies, such as investigating the efficacy of targeted immunomodulatory interventions to enhance the immune response and reduce the incidence of post-stroke pneumonia. Additionally, studies of the optimal timing and intensity of rehabilitation interventions, particularly those addressing respiratory muscle strength, can provide valuable insights for tailored care plans. Research should also focus on better understanding of the impact of pneumonia on neuroplasticity and the intricate interplay between systemic inflammation and neurological recovery. There is also a need to study long-term consequences of SAP on cognitive function and quality of life of stroke patients. Development and implementation of standardized protocols for pneumonia prevention and management in stroke care settings are essential for enhancing overall patient outcomes. Collaborative efforts across multidisciplinary teams and the integration of innovative technologies in rehabilitation and monitoring can further advance our understanding and improve clinical outcomes in this group of patients.

### Limitations:

It includes the inherent heterogeneity among studies, variations in study designs and sample sizes may influence the robustness of the observed associations. While efforts were made to conduct sensitivity analyses and subgroup analyses, the potential impact of unexplored sources of heterogeneity or other confounding variables cannot be entirely ruled out. The diverse range of stroke types, severity and comorbidities within the selected studies may contribute to variability in outcomes and limit the ability to draw generalized conclusions. Variability in diagnostic criteria for post-stroke pneumonia, outcome measures and the duration of follow-up can impact the comparability of results and introduce uncertainties in the synthesis of findings. Furthermore, the reliance on observational studies may have exposed the review to confounding factors and biases inherent in such study designs.

## CONCLUSION

This review provides evidence on the adverse impact of SAP on survival and functional outcomes of stroke patients. The synthesis of evidence calls for a holistic approach to stroke management that recognizes the significance of respiratory complications and uses specific interventions to mitigate their adverse effects. Moving forward, continued research efforts and collaborative initiatives are essential to deepen our understanding, refine risk stratification and improve the overall care and outcomes for stroke patients.

### Authors’ contributions:

**YW:** Literature search, study design and manuscript writing.

**YW and TS:** data collection, data analysis and interpretation. Critical reiew.

**YW:** manuscript revision and validation and is responsible for the integrity of the study. All authors have read and approved the final manuscript.
